# Therapeutic Potential of Stem Cells Strategy for Cardiovascular Diseases

**DOI:** 10.1155/2016/4285938

**Published:** 2016-10-18

**Authors:** Chang Youn Lee, Ran Kim, Onju Ham, Jihyun Lee, Pilseog Kim, Seokyeon Lee, Sekyung Oh, Hojin Lee, Minyoung Lee, Jongmin Kim, Woochul Chang

**Affiliations:** ^1^Department of Integrated Omics for Biomedical Sciences, Graduate School, Yonsei University, 50 Yonsei-ro, Seodaemun-gu, Seoul 120-759, Republic of Korea; ^2^Department of Biology Education, College of Education, Pusan National University, Busan 609-735, Republic of Korea; ^3^Institute for Bio-Medical Convergence, Catholic Kwandong University International St. Mary's Hospital, Incheon 404-834, Republic of Korea; ^4^Department of Neurology and Neurological Sciences, Stanford University School of Medicine, Stanford, CA 94305, USA; ^5^Department of Pharmacology, Yale University School of Medicine, New Haven, CT 06520, USA; ^6^Department of Molecular Physiology, College of Pharmacy, Kyungpook National University, Daegu 702-701, Republic of Korea; ^7^Department of Life Systems, Sookmyung Women's University, 52 Hyochangwon-gil, Yongsan-gu, Seoul 140-742, Republic of Korea

## Abstract

Despite development of medicine, cardiovascular diseases (CVDs) are still the leading cause of mortality and morbidity worldwide. Over the past 10 years, various stem cells have been utilized in therapeutic strategies for the treatment of CVDs. CVDs are characterized by a broad range of pathological reactions including inflammation, necrosis, hyperplasia, and hypertrophy. However, the causes of CVDs are still unclear. While there is a limit to the currently available target-dependent treatments, the therapeutic potential of stem cells is very attractive for the treatment of CVDs because of their paracrine effects, anti-inflammatory activity, and immunomodulatory capacity. Various studies have recently reported increased therapeutic potential of transplantation of microRNA- (miRNA-) overexpressing stem cells or small-molecule-treated cells. In addition to treatment with drugs or overexpressed miRNA in stem cells, stem cell-derived extracellular vesicles also have therapeutic potential because they can deliver the stem cell-specific RNA and protein into the host cell, thereby improving cell viability. Here, we reported the state of stem cell-based therapy for the treatment of CVDs and the potential for cell-free based therapy.

## 1. Introduction

Cardiovascular diseases (CVDs) are a major cause of morbidity and mortality that have a significant impact on health care systems and financial and social consequences. It is estimated that CVDs will be responsible for 23.3 million global cardiovascular deaths worldwide in 2030 [1]. CVDs include diseases that have different causes and characteristics [2]. There is a state of acute conditions such as myocardial infarction and chronic diseases induced by genetic mutations [3]. Classic treatments of CVDs, such as physical surgery and medicinal treatment, are not sufficient to recover damaged cardiovascular tissue and instead only delay the progression of CVDs.

Stem cells, including embryonic stem cells (ESCs), adult stem cells, and induced pluripotent stem cells (iPSCs), are useful in the field of regenerative medicine because they have pluripotency and self-renewal. In addition, stem cells have beneficial effects such as paracrine effects [4, 5], anti-inflammatory activity [6, 7], and immunomodulatory capacity [8]. However, transplantation of stem cells for treatment of heart disease is hampered by their potential for differentiation into host cell types, such as cardiac cells or blood vessel cells, to hinder cardiac function by causing arrhythmia [9] and their low therapeutic effects and viability under the harsh conditions of damaged tissue [10]. To overcome these problems, establishment of new strategies is needed for priming of stem cells.

Interestingly, it has recently been reported that miRNA, small molecules, and extracellular vesicles can modulate the biological activity of stem cells, such as their survival [11, 12], migration [13], and differentiation [14–16]. According to reports, they have been attracting attention owing to their potential to overcome the limitations associated with stem cell therapy. Moreover, a previous study of miRNA small molecules and extracellular vesicles demonstrated that they were effective in the treatment of CVDs. Based on these studies, the stem cell therapy can contribute to treatment of CVDs.

In this review, we describe the state of stem cell therapy for the treatment of CVDs and evaluate the potential for the use of miRNA or stem cell-derived extracellular vesicles.

## 2. Cardiovascular Diseases and Stem Cells

### 2.1. Cause of CVDs

CVDs include a range of major clinical disease conditions, such as cardiomyopathy, hypertensive heart failure, valvular heart disease, peripheral arterial disease, and coronary artery disease [25, 26]. Senescence and male typically raise hazards of CVDs and are primary criteria used to classify risk evaluation [27, 28]. CVDs are strongly connected to lifestyle, age, and gender, with alcohol and tobacco consumption, physical inactivity, psychosocial factor, diet and obesity, and increased blood pressure being major factors [28]. In addition, psychosocial risk elements including low socioeconomic status, deficiency of social support, stress, depression, anxiety disorder, and hostility contribute to development of CVDs [28–34]. Familial prevalence of CVDs is another major risk factors [28]. Nutrient deficiency including low vitamin D levels also plays a role in CVDs pathogenesis [35]. Several studies have shown that vitamin D affects heart function through regulation of hormonal systems including the parathyroid hormone and renin-angiotensin system [36, 37]. Vitamin D has also been shown to affect the cell cycle of cardiomyocytes, to inhibit cardiac cell proliferation, and to protect the structure and function of cardiomyocytes [35, 38, 39]. Hypertension is the most common CVD, leading to a growing risk of stroke, myocardial infarction, and heart and renal failures [40]. Previous clinical tests have indicated that decreased blood pressure reduces the outbreak of CVDs such as stroke and heart attack [40]. There have been various efforts to overcome CVDs in the past few decades [41]. Despite advanced medical and surgical trials, there are still no effective therapies for treatment of CVDs [42, 43]. Unlike other organs, the recuperative ability of heart is limited for treatment of injured cardiac tissue [43]. Heart transplantation can be utilized as a last resort to treat CVDs, but the approach is expensive and extremely limited for patients because of their comorbidities or poor supply of donor organs [43].

### 2.2. Embryonic Stem Cell Therapy for CVDs

Recent studies have suggested that stem cell therapies target cardiac regeneration in CVDs [42, 43]. Application of stem cells to therapeutic devices and methods may lead to effective and rapid myocardium regeneration and eventually affect cardiovascular morbidity and mortality [44]. There are different types of stem cells such as embryonic stem cells, adult stem cells, and induced pluripotent stem cells for treatment of CVDs (Table 1). Previous studies have demonstrated therapeutic effects of ESCs differentiated into cardiomyocytes [17] and endothelial cells [18] for myocardial infarction, umbilical-cord-blood-derived MSCs for dilated cardiomyopathy [19], bone-marrow-derived-MSCs for cellular cardiomyoplasty [20], and iPSCs [21] and iPSCs-derived cardiovascular progenitor cells [22], endothelial progenitor cells (EPCs) [23], and cardiac stem cells [24] for myocardial infarction.

#### 2.2.1. Therapeutic Characteristics of ESCs

ESCs are pluripotent cells derived from the inner cell mass and infinitely replicate without senescence, maintaining their undifferentiated state [45]. This cell population is called ESCs and follows irreversible process of differentiation to become specialized [44, 46]. ESCs have pluripotency, with the capacity of differentiation into approximately 210 different cell types, making them an up-and-coming stem cell source for cell-based tissue engineering [44]. ESCs can be differentiated into cardiomyocytes, endothelial cells, and vascular smooth muscle cells [17, 18, 47, 48]. Owing to their potential for use in the treatment of CVDs, efforts for stem cell therapy have been concentrated on the differentiation of human ESCs into the cardiac lineage directly [44, 49].

#### 2.2.2. Using ESCs for Myocardial Regeneration Therapy

In previous studies, undifferentiated ESCs or ESCs differentiated into cardiomyocytes, endothelial cells, or vascular cells were directly injected into animal CVDs models [17, 18, 50]. Treatment with those ESC-derived cells showed beneficial effects such as improvement of cardiac regeneration and remodeling and increased myocardial performance [1, 17, 18, 50]. Therapeutic effects of the differentiated cells are mediated through cell engraftment and proliferation and paracrine effects [1, 51, 52]. Recent studies have suggested that genetic and epigenetic regulations of cardiomyocytes differentiation are new approaches to inducing a cardiac lineage for stem cell therapy [43]. Deletion or knockdown of microRNA (miRNA), a small regulator of gene expression, brings about dysregulation of morphogenesis, electrical conduction and hypertrophy of heart, and the cell cycle of cardiocytes [14, 43, 53]. Ivey et al. demonstrated that miR-1 and miR-133 can control the ability to differentiate into the cardiac lineage in mouse and human ESCs [53]. Moreover, epigenetic modification can regulate ESCs differentiation and genetic control [43]. Weber et al. suggested that histone deacetylation is involved in cardiovascular development through target regulation of hey bHLH as major effector in Notch signaling [54]. Although ESCs have considerable potential for direct differentiation into cardiac lineage in various models, several limitations hinder their clinical applications [41]. The greatest limitation is that research using ESCs is hampered by ethical issues that prevent their actual clinical applications [41, 55]. Moreover, treatment with ESCs poses the risk of immunorejection because of differences in genome information among patients [41, 56].

### 2.3. Adult Stem Cell Therapy for CVDs

#### 2.3.1. Therapeutic Characteristics of MSCs

In adult bodies, tissues and organs contain a small cell subpopulation with the capacity for self-maintenance through the potential to proliferate indefinitely and the ability to form an extended family of daughter cells [57, 58]. These cells are widely known as adult stem cells [7]. Mesenchymal stem cells (MSCs) are found in most of adult tissues, including bone marrow and adipose tissues [58, 59]. The nonhematopoietic cells can be differentiated and modified* in vitro* to present phenotypes of cardiomyocytes and vascular endothelial cells [58]. In addition, MSCs are able to produce and secrete a broad variety of cytokines, chemokines, and growth factors for enhancing neovascularization, attenuating fibrosis in heart, and recovering cardiac functions [4, 5, 60]. Accordingly, it is possible that MSCs could be a therapeutic cell source with the capacity to repair injured tissue in CVDs.

#### 2.3.2. Treatments of Myocardial Diseases by Using MSCs

Bone-marrow-derived MSCs (BM-MSCs) have been widely reported as a promising therapeutic strategy for CVDs [61]. These cells can differentiate into cardiomyocytes and endothelial cells [20, 61]. Many studies have indicated that BM-MSCs possess therapeutic effects in heart diseases such as myocardial infarction, diabetic cardiomyopathy, and dilated cardiomyopathy [61–63], and BM-MSCs are now considered one of the most attractive adult stem cell populations for cardiovascular repair [61, 64]. Cai et al. showed that BM-MSCs cocultured with neonatal rat ventricular cardiomyocytes prevented isoproterenol-induced typical hypertrophic characteristics of cardiomyocytes in* in vitro* and* in vivo* studies [61]. Moreover, interplay of BM-MSCs with cardiomyocytes produced synergistic effects on VEGF secretion [61]. Today, many studies showed that paracrine factors, such as VEGF, bFGF, and IGF-1, play an essential therapeutic role [4]. Tang et al. demonstrated that autologous BM-MSCs transplantation in rat MI model improved vascular regeneration and cardiac performance through paracrine effect of VEGF, bFGF, and SDF-1 [5]. Ohnishi et al. also found conditioned medium of BM-MSCs affected the antiproliferation of cardiac fibroblasts via expressing paracrine antifibrotic effects of MMPs [60]. Adipose tissue-derived MSCs (AD-MSCs) have become an attractive therapeutic cell source [65] because they are easily expanded* in vitro* and express the same cell surface markers as BM-MSCs [65, 66]. Moreover, injected AD-MSCs have been shown to differentiate toward a cardiogenic phenotype [67] and reduce the infracted size, exhibiting a powerful and persisting angiogenic potential [65, 68]. Siciliano et al. reported on plasticity of human AD-MSCs and their phenotypical modification in cardiac-specific microenvironments [65]. They also indicated that human AD-MSCs cocultured with cardiospheres-conditioned media changed toward a cardiac/endothelial/muscular-like phenotype in response to regulation of the expression of cardiogenic markers and induction of the activation of intracellular survival signaling pathways [65]. Umbilical-cord-blood-derived MSCs (UCB-MSCs) are a new xenogeneic stem cell therapy source for CVDs [19]. The newly proposed cell source may be optimum for CVDs because they have a low immunogenicity and a large change of cardiomyocyte reprogramming of UCB-MSCs in comparison with xenogeneic stem cells [19, 69–71]. In addition, UCB-MSCs are easily obtained through low-invasive surgery without raising ethical issues, demonstrating their promising clinical application [19]. Gong et al. demonstrated that intramyocardial grafts of human UCB-MSCs promote cardiac function via mechanisms of antiapoptosis, anti-inflammation, and proangiogenesis in cardiomyopathy of cTnT^R141W^ transgenic mice [19]. They also found that UCB-MSCs derived conditioned medium protects H9C2 cells from apoptosis in hypoxic condition by paracrine effects* in vitro* [19].

#### 2.3.3. Treatments of Myocardial Diseases by Using Other Stem Cells (CPCs, EPCs)

The tissue-specific stem cells were found in several tissues, such as skeletal muscle, brain, fat, liver, gastrointestinal tract, and epidermis. These stem cells are differentiated into specific tissue cells and contribute to maintaining tissue homeostasis. Interestingly, the tissue-specific stem cells were found in heart [72]. Heart was not known to be able to self-regenerate before the establishment of cardiac progenitor cells (CPCs). Since then, cardiac progenitor cells have been reported on therapeutic potential in CVDs.

Beltrami et al. reported on Lin^−^ c-Kit^POS^ cells in CPCs. They are self-renewing and multipotent and have colony forming ability [72]. Injected CPCs into the ischemic heart can be differentiated into cardiomyocytes, reconstruct heart, and induce new blood vessel. They suggest that CPCs to repair the heart provide a new opportunity. However, CPCs present in very small amounts in the heart and require* in vitro* expansion of a few weeks [73].

In 1997, Asahara et al. reported that isolated CD34^POS^ cells are endothelial progenitor cells which are separate from peripheral blood [74]. EPCs also have capacity of differentiation into endothelial cells and angiogenesis. For that reason, EPC was noted in the study for treating various ischemic injury. Kawamoto et al. studied effects of heart regeneration by transplantation of EPCs into rat MI heart in 4 weeks after transplantation [23]. According to this paper, the effect of regeneration is caused by angiogenesis which is promoted by transplanted EPCs into ischemic damage area.

Another strategy is reported based on the stimulation of the EPCs* in vivo* for treating ischemic disease. Oikonomou et al. reported that 26 patients with heart disease have improved blood vessel function after administration of atorvastatin for 4 weeks by increasing circulated EPCs [75].

Transplantation of EPC has benefit for treatment of disease, but we need to concern about immune rejection in the allograft because therapy using EPCs is based on the autograft method. Therefore, we need to overcome this limitation for transplantation into damaged area.

### 2.4. iPSCs as a Source of Cell Therapies for CVDs

In 2006, iPSCs were established by Takahashi and Yamanaka by reprogramming mouse fibroblasts through overexpression of four specific transcription factors: Oct3/4, Sox2, c-Myc, and Klf4 [76]. Since this breakthrough, various opportunities for application have been reported in cell therapy, modeling of new diseases, and studies of complex genetic features and allelic variations, as substrates for drug, in toxicity, differentiation, and regenerative medicine therapies, and in therapeutic screening [77, 78]. In addition, using the iPSCs, it is possible to differentiate into patient- and disease-specific cell types. Thus, using iPSC is one of the tools for treatment of the patients [79, 80]. However, many considerations, including the optimal materials for reprogramming, high cost, safety, and efficient derivation, still remained regarding using iPSCs for clinical applications [81]. Thus, it is essential to understand the following events after the activation of reprogramming factors for transplantation of iPSCs for a safe way.

#### 2.4.1. Methods for Differentiation from iPSCs to Cardiomyocytes

**Figure 1:**
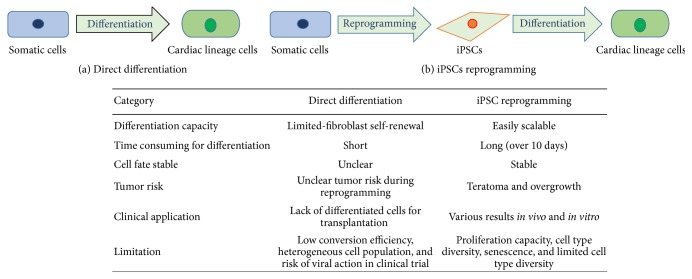
Strategies for differentiation from somatic cells into cardiac lineage cells.

**Figure 2:**
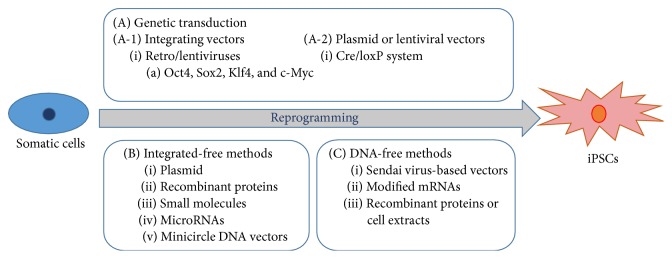
Two ways for inducing pluripotent cells from somatic cells.

Recently various efficient methods for inducing differentiation into cardiac lineage cells using iPSCs or direct reprogramming (Figure 1). iPSCs have been tried to use genetic transduction or integrated-free methods. Using viral vector for reprogramming has higher efficiency than using integrated-free methods, but safety is lower than using integrated-free methods (Figure 2) [82]. Another method for differentiation into cardiac lineage cells is direct differentiation. One of the protocols for direct differentiation is overexpression of combined cardiac-specific transcription factors such as GATA4, Mef2c, and Tbx5 (GMT) [83, 84] or GMT and Hand2 [85]. Another method is using miRNAs or small molecules without lineage-specific transcription factors: overexpression of miRNAs 1, 133, 208, and 499 is effective to increase the capacity of direct differentiation into cardiac lineage cells [86]: chemically defined medium (CDM) containing three components is also reported that can induce differentiation into cardiomyocytes [87]. Recently, Hou et al. also reported that treatment of seven small molecules into cells can generate iPSCs [88]. Direct differentiation into cardiac-specific lineage may provide the therapeutic strategy for cardiac regeneration; however, it needs to improve the low efficiency of differentiation into the cardiac lineage cells [84].

#### 2.4.2. Treatment of CVDs by Transplanted iPSCs

iPSCs have characters like embryonic stem cells (ESCs)—pluripotency and self-renewal—so that iPSCs can be provided to be transplanted into damaged tissue or organ [89]. Masumoto et al. reported cardiac regeneration by transplantation of engineered human iPSC as a cardiac tissue sheet (hiPSC-CTSs) into rat heart [90]. They checked that heart function was improved and survival of transplanted cells was over 40% cells. Rojas et al. performed transplantation of iPSCs with fibrinogen into heart of myocardial injury model [21]. Although heart function was recovered by transplantation of iPSCs into damaged heart, they suggested transplantation of iPSC-derived cardiomyocytes is more relevant for clinical trial. In addition, the problems of transplantation of iPSCs including tumorigenicity, immunogenicity, and genomic instability have been reported [88, 91–94]. Recently, transplantation of differentiated cells into tissue-specific lineage is reported to overcome the aforementioned problems; Funakoshi et al. reported transplantation of iPSC-derived cardiomyocyte and they tried to optimize condition for transplantation [95]; and Ja et al. showed the effects of the transplantation of iPSC-derived human cardiac progenitor cells improved heart function [22]. They suggested that the improvement of heart function is caused by angiogenesis and interstitial networking of damaged heart.

### 2.5. Cell-Free Therapeutic Strategy for CVDs

Extracellular vesicles have been reported to modulate a variety of biological actions in the cells, such as proliferation, migration, apoptosis, and differentiation. Stem cell-derived extracellular vesicles especially have potent cardiac protection, regeneration, and angiogenic properties. In addition to affordable benefits, it is well known the vesicles can be used to communicate with other cells and control level of protein expression. Thus, transplantation of extracellular vesicles has the potential as a novel cell-free therapy for treatment of CVDs, which has various advantages to overcome the limitations related to the cell-based therapeutics.

#### 2.5.1. Treatment of CVDs by Extracellular Vesicles-Derived Stem Cells

Various reports have emphasized the effectiveness of secretion factors during treatment of heart disease using stem cells. Paracrine effect is one of the benefits by transplantation of stem cells into the damaged area causing secretion of various cytokines and growth factors [4, 5]. Recently, many studies have investigated cell-derived extracellular vesicles, and they have been shown to exert positive effects on a variety of cellular activities such as antiapoptosis, migration, differentiation, and cell recruitment [96]. In fact, extracellular vesicles have a biologic function of communication between the cells and recipient cells. Several studies have reported that the extracellular vesicles contained various factors including nucleic acids, cytokines, growth factor, and miRNAs [97–99]. Lai et al. reported that extracellular vesicles can promote repair of damaged heart by myocardial ischemia/reperfusion injury [100]. They have demonstrated that the exosome was contained in the stem cell conditioned media so that it contributed to the cardiac protection. Lee et al. demonstrated that mesenchymal stromal cell-derived exosomes (MEX) inhibited vascular remodeling and hypoxic pulmonary hypertension [101]. They also demonstrated that MEX inhibit the inflammatory response and cell proliferation of vascular pulmonary hypertension in animal models. They suggested that the reason of the advantages was based on the downregulation of miR-17 cluster and stat3 signaling in MEX-treated vessel cells. Khan et al. reported that ESC-derived exosome promoted the repair of ischemic myocardium [102]. Therapeutic potential of ESC-derived exosome stimulates CPCs activity including survival, cell cycle progression, and proliferation, by overexpression of ESC-specific miR-294. Furthermore, therapeutic effects of CPCs-derived exosomes have been reported [103]. Vrijsen et al. reported that CPCs-derived exosomes promote cell migration* in vitro* wound assay [104]. According to their report, CPCs-derived exosomes include EMMPRIN and MMP, to induce endothelial cell migration. Chen et al. reported a myocardial infarction protective effect of the CPCs-derived exosome [105]. CPCs-derived exosome contains higher level of miR-451 so it can protect H9C2 cells from oxidative stress by inhibiting caspase-3 and caspase-7 activation.

Although there are a lot of experiments that have studied transplantation of extracellular vesicles which have beneficial effect in treating CVD* in vitro* and* in vivo*, we still need to consider side effects on other organs, appropriate amount for transplantation, and compatibility for clinical practices in human. Therefore, more researches which targeted safety to use for therapeutic approaches using extracellular vesicles are required in future clinical trials.

### 2.6. Limitations of Stem Cell and Cell-Free Therapy Strategies for CVDs

Stem cell therapy should be more considered for efficient clinical application in CVDs. Despite the many positive efforts for stem cell therapy, it has still some problems, such as low efficiency, immune rejection, and difficulty in control of stem cell behavior* in vivo*. In addition, administered stem cells often do not show effective integration or persistence in the heart tissues and trigger tumor formation [106].

On the other hand, the cell-free therapy was proposed as a means to avoid such a problem which can occur in stem cell therapy. However, the short half-life is a problem of the cell-free therapy because the proteins and nucleic acids are rapidly biodegradable* in vivo* as a main component [107–109]. For this reason, cell-free therapy must be administered more frequently. Moreover, disease specificity, biodistribution, and persistency of the cell-free factors must be validated before clinical application [110].

## 3. Conclusions

In this review, we focus on the strategy for treatment of CVDs by transplantation of stem cells. Transplantation of stem cells including ESCs, adult stem cells, and iPSCs can promote tissue regeneration of damaged area. Stem cells have specific characters such as self-renewal and pluripotency. In addition, stem cells secrete paracrine factors, so transplantation of which shows beneficial effects. Stem cell-derived vesicles can stimulate the cell activity by transferring beneficial materials, including the stem cell-specific transcription factor and miRNA to the damaged tissue. Thus, transplantation of vesicle can contribute to recovery in damaged area. In addition, treatment of additional materials such as miRNA or small molecules for promoting differentiation of stem cells into specific cell types can improve therapeutic effects for treatment of CVDs.

Although stem cell therapy has problems such as low survival rate after transplantation into harsh condition and still needs to overcome these problem, stem cell therapy and cell-free based therapy have potential to improve heart function after CVDs.

## Figures and Tables

**Table 1:** Application of stem cells for therapy of CVDs.

Disease model	Stem cell type	Delivery route	Dose and follow-up duration	Outcomes	Reference
Rat MI	ESC-CMs	Intramyocardial transfer	1.5 × 10^6^ 8 weeks	Observation of grafted cardiomyocytes survival, proliferation, maturation, alignment, and forming gap junctions with host cardiac tissue	[17]

Mouse MI	ESC-ECs	Intramyocardial transfer	1 × 10^6^ 8 weeks	Appropriate patterns of endothelial gene expression, functional vessels formation *in vivo*, and cardiac function improvement	[18]

Mouse DCM	UCB-MSCs	Intramyocardial transfer	1.5 × 10^6^ 4 weeks	Improvement of cardiac function by antiapoptosis, anti-inflammation, and proangiogenesis	[19]

Mouse cellular cardiomyoplasty	BM-MSCs	Intramyocardial transfer	5–10 × 10^6^ 4–8 weeks	Engrafted hMSCs from adult BM in the myocardium to differentiate into cardiomyocytes	[20]

Mouse MI	iPSCs	Intramyocardial transfer	1 × 10^5^ 2 weeks	Improved iPSCs maintenance through improved function and cell proliferation in infarcted myocardium	[21]

Mouse MI	iPSC-CPCs	Intramyocardial transfer	2 × 10^5^ 2–4 weeks	Exertion of protective effect on LV remodeling by paracrine effects through enhanced angiogenesis and augmented networking in the infarcted milieu	[22]

Rat MI	EPSs	Intramyocardial transfer	1 × 10^6^ 7 weeks	Increase of regional wall motion and decrease of ventricular dimension in left ventricle	[23]

Rat MI	CSCs	Intramyocardial transfer	5 × 10^6^ 2–4 weeks	Reduction of ejection fraction, fractional shortening, and infracted size of the left ventricle	[24]

BM: bone marrow; CMs: cardiomyocytes; CPCs: cardiovascular progenitor cells; CSCs: cardiac stem cells; DCM: dilated cardiomyopathy; ECs: endothelial cells; EPCs: endothelial progenitor cells; ESC: embryonic stem cell; h: human; iPSC: induced pluripotent stem cell; MI: myocardial infarction; MSCs: mesenchymal stem cells; and UCB: umbilical cord blood.
